# Characterization of bacterial communities associated with the pinewood nematode insect vector *Monochamus alternatus* Hope and the host tree *Pinus massoniana*

**DOI:** 10.1186/s12864-020-6718-6

**Published:** 2020-05-01

**Authors:** Yajie Guo, Qiannan Lin, Lyuyi Chen, Rebeca Carballar-Lejarazú, Aishan Zhang, Ensi Shao, Guanghong Liang, Xia Hu, Rong Wang, Lei Xu, Feiping Zhang, Songqing Wu

**Affiliations:** 1 College of Forestry, Fujian Agriculture and Forestry University, Fuzhou, 350000 China; 2 Key Laboratory of Integrated Pest Management in Ecological Forests, Fujian Province University, Fujian Agriculture and Forestry University, Fuzhou, 350000 China; 3 State Key Laboratory of Ecological Pest Control for Fujian and Taiwan Crops, Fujian Agriculture and Forestry University, Fuzhou, 350000 China; 4 Universityof California, Irvine, CA 92697-4025 USA; 5 Department of Microbiology & Molecular Genetics, University of California, Irvine, CA 92697-4025 USA; 6 Graduate School of Chinese Academy of Agricultural Sciences, Beijing, 100081 China

**Keywords:** *Monochamus alternatus* Hope, *Pinus massoniana*, Microbial community, 16S rDNA, Diversity analysis

## Abstract

**Background:**

*Monochamus alternatus* Hope is one of the insect vectors of pinewood nematode (*Bursaphelenchus xylophilus*), which causes the destructive pine wilt disease. The microorganisms within the ecosystem, comprising plants, their environment, and insect vectors, form complex networks. This study presents a systematic analysis of the bacterial microbiota in the *M. alternatus* midgut and its habitat niche.

**Methods:**

Total DNA was extracted from 20 types of samples (with three replicates each) from *M. alternatus* and various tissues of healthy and infected *P. massoniana* (pines). 16S rDNA amplicon sequencing was conducted to determine the composition and diversity of the bacterial microbiota in each sample. Moreover, the relative abundances of bacteria in the midgut of *M. alternatus* larvae were verified by counting the colony-forming units.

**Results:**

Pinewood nematode infection increased the microbial diversity in pines. *Bradyrhizobium*, *Burkholderia*, *Dyella*, *Mycobacterium*, and *Mucilaginibacter* were the dominant bacterial genera in the soil and infected pines. These results indicate that the bacterial community in infected pines may be associated with the soil microbiota. Interestingly, the abundance of the genus *Gryllotalpicola* was highest in the bark of infected pines. The genus *Cellulomonas* was not found in the midgut of *M. alternatus*, but it peaked in the phloem of infected pines, followed by the phloem of heathy pines. Moreover, the genus *Serratia* was not only present in the habitat niche, but it was also enriched in the *M. alternatus* midgut. The colony-forming unit assays showed that the relative abundance of *Serratia* sp. peaked in the midgut of instar II larvae (81%).

**Conclusions:**

Overall, the results indicate that the bacterial microbiota in the soil and in infected pines are correlated. The *Gryllotalpicola* sp. and *Cellulomonas* sp. are potential microbial markers of pine wilt disease. Additionally, *Serratia* sp. could be an ideal agent for expressing insecticidal protein in the insect midgut by genetic engineering, which represents a new use of microbes to control *M. alternatus*.

## Background

Pine wilt disease is a destructive disease of pine trees caused by the pinewood nematode, *Bursaphelenchus xylophilus* (Steiner & Buhrer) Nickle, which causes significant environmental and economic losses worldwide [1]. It originated in North America and then spread to Asia and Europe [2, 3]. In Japan, pine wilt disease has threatened pine forests since 1905, with the loss of 700,000 m^3^ of pine trees each year [3, 4]. In China, since the discovery of pinewood nematode in Nanjing in 1982, the disease has spread rapidly, threatening the safety of nearly 60 million hectares of pine trees. In Asia, pinewood nematode infection mainly occurs during feeding and oviposition of adults of the beetle species known as *Monochamus alternatus* Hope, which spreads the disease among pine trees [5, 6]. Therefore, effective prevention and control of *M. alternatus* populations are one of the best approaches to control pine wilt disease.

Microbial insecticides, the most widely used biological control method, have not been well developed for controlling wood-boring insects such as *M. alternatus* [7–10]. The main problem to overcome is that it is difficult for the insecticidal protein to enter the tree trunk to reach the *M. alternatus* larvae [7–10]. However, research has shown that mosquitoes can become resistant to malaria infection as a result of colonization by symbiotic bacteria carrying antimalaria effector molecules to the mosquito midgut lumen [11]. A study has shown that a strain of *Serratia* bacteria (AS1) can colonize the mosquito midgut and inhibit the growth of the malaria parasite *Plasmodium falciparum* in mosquitos [12]. Therefore, the purpose of this study was to identify a bacterial species that is present in the habitat niche and is enriched in the midgut of *M. alternatus* larvae, as this species could potentially be used as a carrier of an insecticidal protein that is toxic to *M. alternatus* larvae.

The microbiomes in plants, insects and soil make up an aboveground-belowground microbiota environment, it has become a hotspot to study the role of changes in these microbiomes in theses interactions [13–15]. Many studies have investigated the associations between the bacterial communities of pinewood nematode, pine trees, and insect vectors, including various instars of *M. alternatus* [16]; *M. alternatus* and *M. galloprovincialis* adults [17, 18]; *M. galloprovincialis* and pinewood nematode [1, 19–27]; *Pinus* trees [28–31]; pinewood nematode and infected *Pinus* trees [32, 33]; pinewood nematode, infected *Pinus pinaster* trees, and the vector *M. galloprovincialis* [34]; and the soil of infected *Pinus* trees [35]. However, few studies have analyzed the associations between the bacterial communities of the insect vector *M. alternatus*, host tree *P. massoniana* (pines), and soil.

In this study, *M. alternatus*, pines, and soil were systematically sampled from the same location during the same time period. The compositions of each microbiota in the *M. alternatus* midgut and its habitat niche were analyzed by 16S rDNA amplicon sequencing. The bacterial communities associated with *M. alternatus* and pines were characterized. The relative abundance of a bacterial species of interest (*Serratia* sp.) in the various instar larvae were verified by conducting colony-forming unit assays. The results contribute to the understanding of the differences among the microbiomes of *M. alternatus* and its habitat niche.

## Results

### Operational taxonomic unit (OTU) sequencing results

**Fig. 1:**
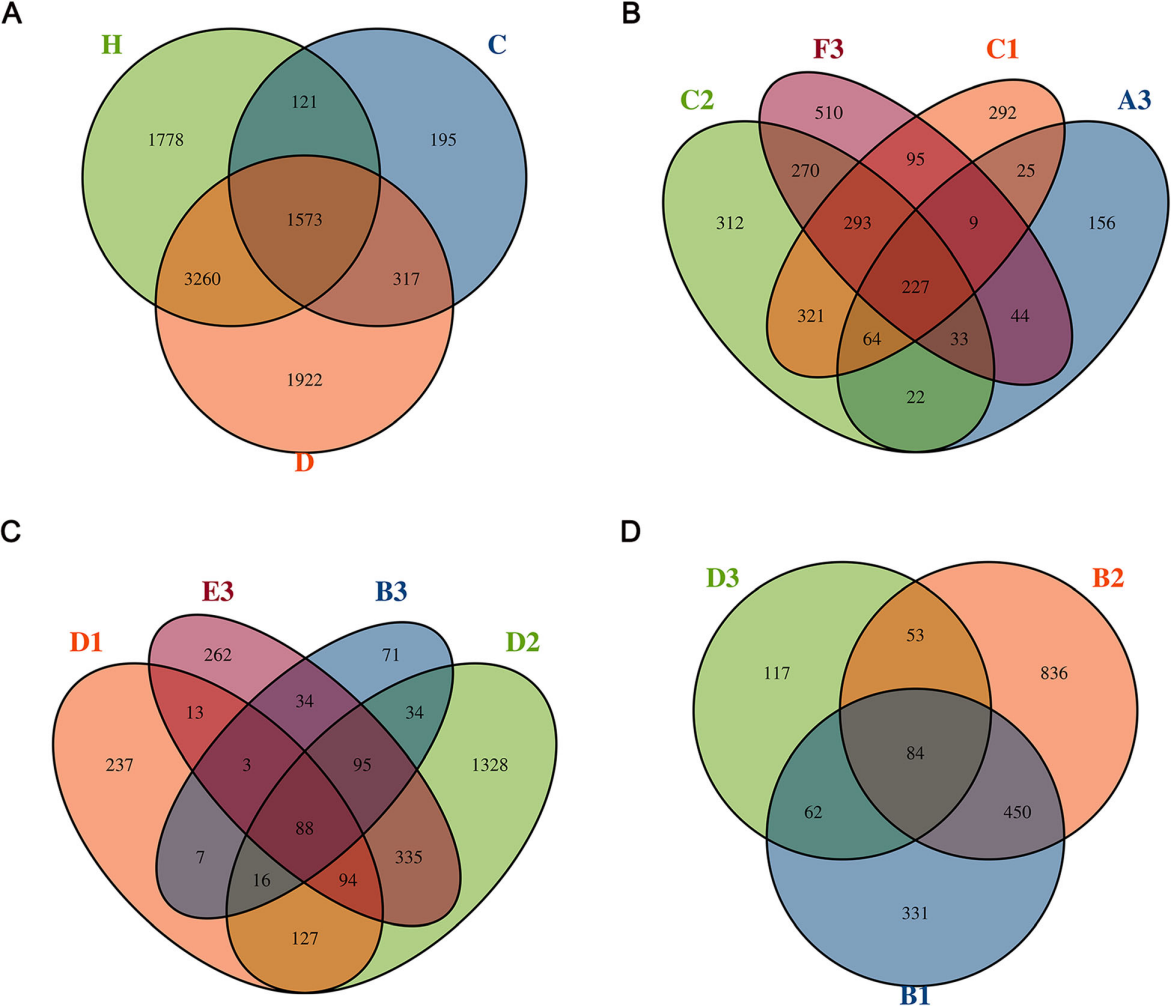
Operational taxonomic unit (OTU) Venn diagrams. (**a**) H: healthy pine (*Pinus massoniana*) and soil, D: infected pine and soil, C:* Monochamus alternatus* and frass; (**b**) A3: instar II larvae midgut, C1: healthy pine phloem, C2: infected pine phloem, F3: instar II larvae frass; (**c**) B3: instar III larvae midgut, D1: healthy pine xylem, D2: infected pine xylem, E3: instar III larvae frass. (**d**) D3: adult midgut, B1: healthy pine bark, B2: infected pine bark

A total of 9174 OTUs were obtained from the 60 samples of *M*. *alternatus* and its habitat niche. According to the rarefaction curves, the number of sequences obtained was able to reflect the main bacterial information in each sample (Additional file 1: Figure S1). There were 1573 OTUs shared among all samples. 1778 and 1922 unique OTUs were detected in samples from healthy and infected pines, respectively. Only 195 unique OTUs were found in samples from *M*. *alternatus* (Fig. 1a)*.* Instar II larvae feed on phloem, and the number of OTUs shared by the instar II larvae midgut and the phloem of infected pine (346) was close to the number shared by instar II larvae midgut and the phloem of healthy pine (325) (Fig. 1b). Instar III larvae feed on xylem, and the number of OTUs shared by instar III larvae midgut and the xylem of infected pine (233) was approximately twice that shared by instar III larvae midgut and the xylem of healthy pine (114). There were 1328 unique OTUs in the xylem of infected pines, which was far more than the 237 unique OTUs in the xylem of healthy pines (Fig. 1c). There were 84 shared OTUs in the samples from midgut of adult *M. alternatus*, healthy pine bark, and infected pine bark. The number of unique OTUs (not found in the adult *M. alternatus*) in infected pine bark was about 2.5 times that in healthy pine bark (Fig. 1d).

### Linear discriminant analysis effect size (LEfSe) analysis

**Fig. 2:**
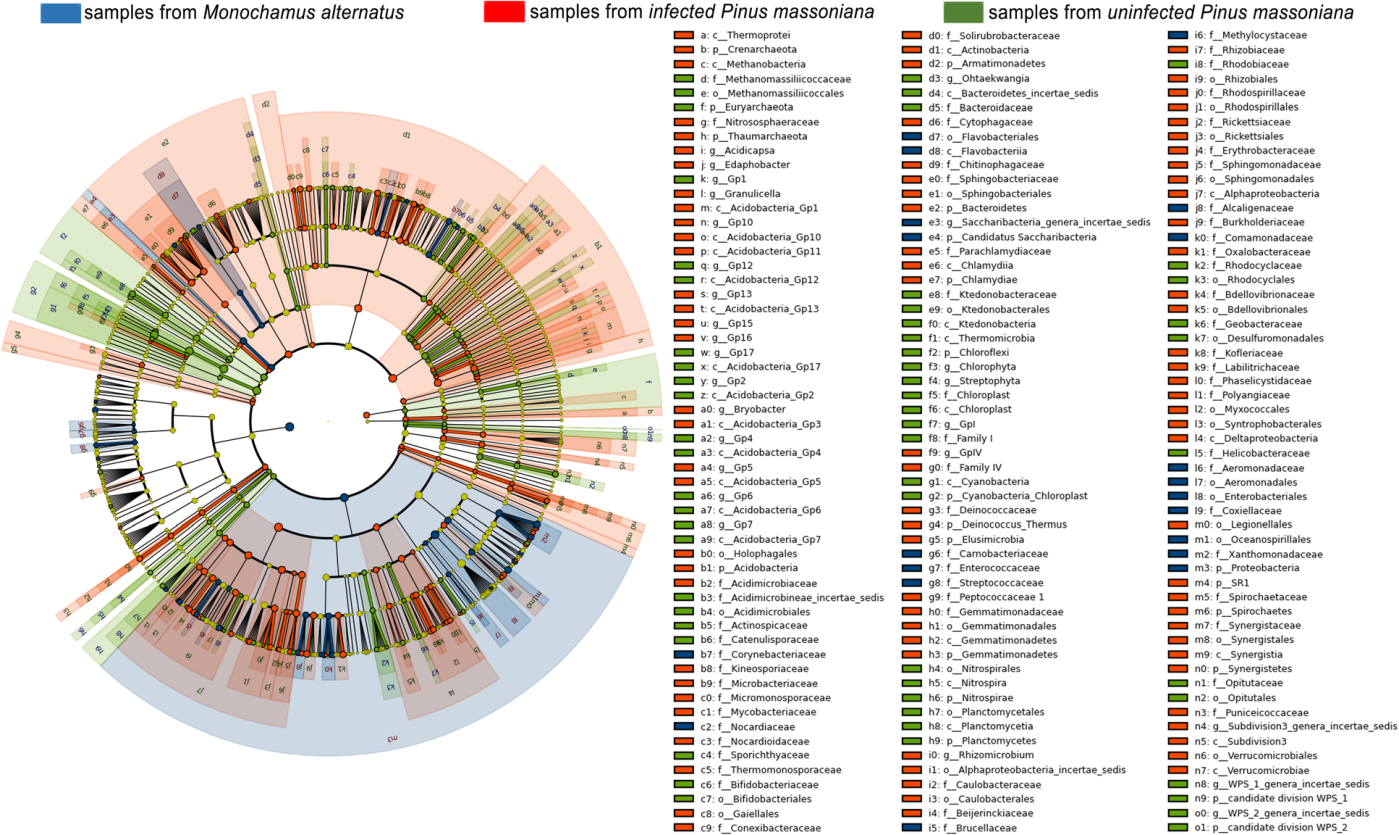
Cladogram of bacteria from *Monochamus alternatus* and infected and healthy *Pinus massoniana*. Different colors represent different bacterial groups, and nodes of different colors represent the bacteria that play important roles in each group, i.e., blue, red, and green nodes represent the bacteria that play important roles in samples from *M*. *alternatus*, infected *P. massoniana*, and healthy *P. massoniana*, respectively*,* while the yellow nodes represent bacteria that do not play important roles

Species distribution analysis at the phylum level indicated that the main bacteria in the *M. alternatus* midgut belonged to Proteobacteria and Firmicutes*.* Infected pines mainly harbored Bacteroidetes, Armatimonadetes, Actinobacteria, Acidobacteria, and Proteobacteria (Additional file 1: Figure S2). The Acidobacteria in infected pines was highly similar to that in healthy pines, while the Proteobacteria in infected pines was highly similar to that in the midgut of *M. alternatus* (Fig. 2).

### Bacterial community compositions in *M. alternatus* and its habitat niche

**Fig. 3:**
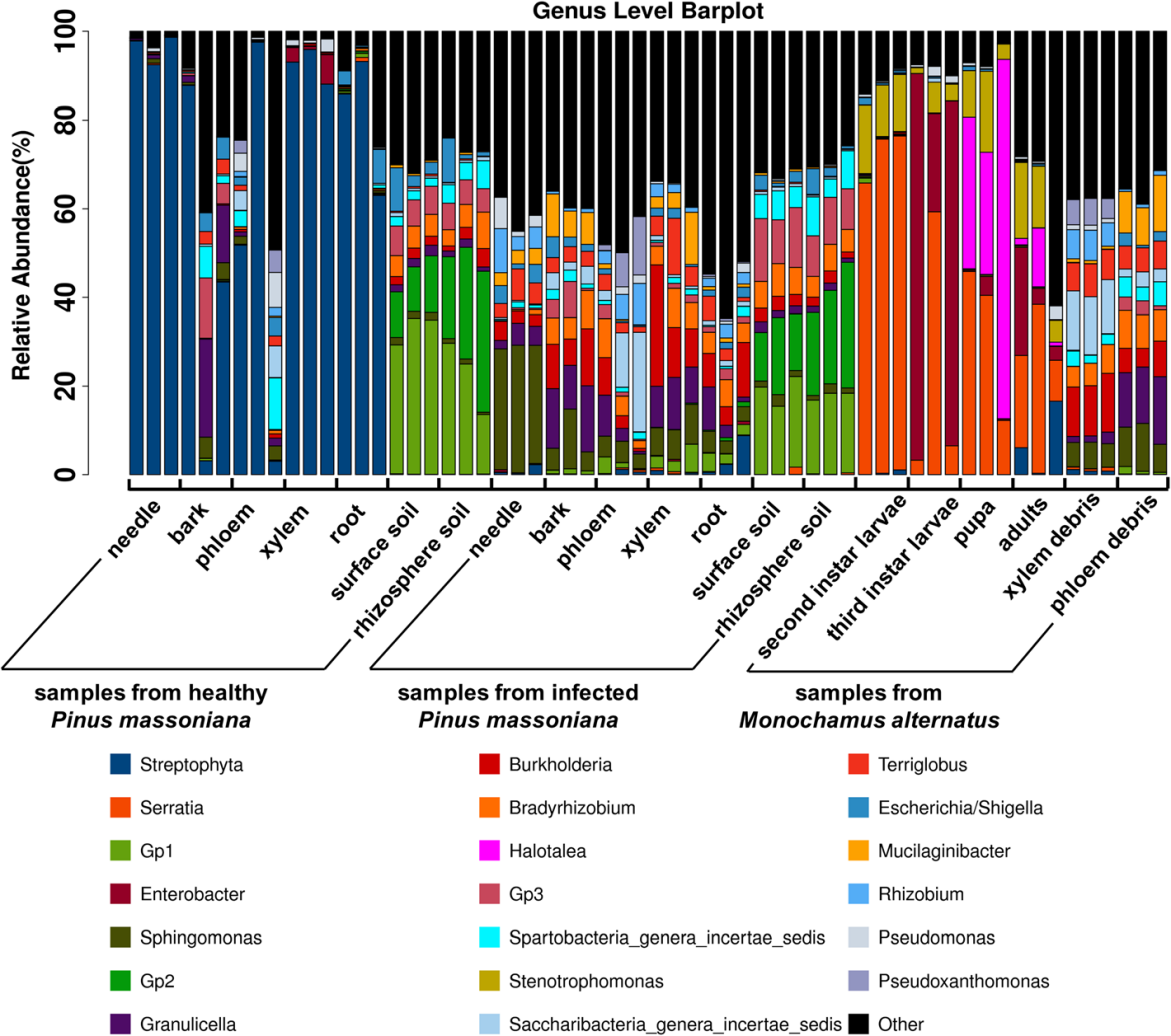
Stacked bar plot of bacterial genera from *Monochamus alternatus* and infected and healthy *Pinus massoniana*. The 20 most abundant OTUs are shown, with the remaining grouped together in the group labeled “other”

There were significant differences in species composition between infected and healthy pines. The *Streptophyta* of Cyanobacteria/Chloroplast was the dominant in healthy pines, due to the V3-V4 region cannot distinguish 16 s rDNA from bacteria and Cyanobacteria/Chloroplast. Regarding the infected pines, the most abundant genera were *Sphingomonas* (7.66%), followed by *Burkholderia* (6.51%) and Acidobacteria subgroup 1 (Gp1) (6.51%). In the midgut and frass of *M. alternatus*, the most abundant genera were *Serratia* (25.25%), *Enterobacter* (12.42%), *Halotalea* (8.81%), and *Stenotrophomonas* (6.68%). The relative abundance of Acidobacteria subgroup 1 (Gp1), subgroup 2 (Gp2), and subgroup 3 (Gp3) in surface soil and rhizosphere soil exceeded 50%, with no differences between infected and healthy pines (Fig. 3) (Additional file 1: Figures S3, S4).

Regarding the frass of different stages of *M. alternatus* after feeding, *Granulicella* was the most abundant genus (12.15%) in the frass of instar II larvae, followed by genus *Sphingomonas* (10.11%). *Saccharibacteria* was the most abundant genus in the frass of instar III larvae (12.57%), followed by genus *Burkholderia* (11.68%). The relative abundance of genus *Pseudoxanthomonas* (5.31%) in the frass of instar III larvae was higher than in the frass of instar II larvae and the midgut of various instars (total: 0.03%) (Fig. 3 and Additional file 1: Figures S5, S6). After feeding by *M. alternatus* adults, the most abundant genera in the bark from infected pines were *Sphingomonas* and *Granulicella* (Additional file 1: Figure S7). The bark, phloem, and xylem of infected pines contained more putative pathogenic bacteria (mainly *Saccharibacteria*, *Burkholderia*, and *Granulicella*) than the corresponding tissues in healthy pines (Fig. 3). These results indicate that the dominant bacteria were similar between the frass of larvae and infected pines.

### Specific bacterial genera in the habitat niche of *M. alternatus*

**Fig. 4:**
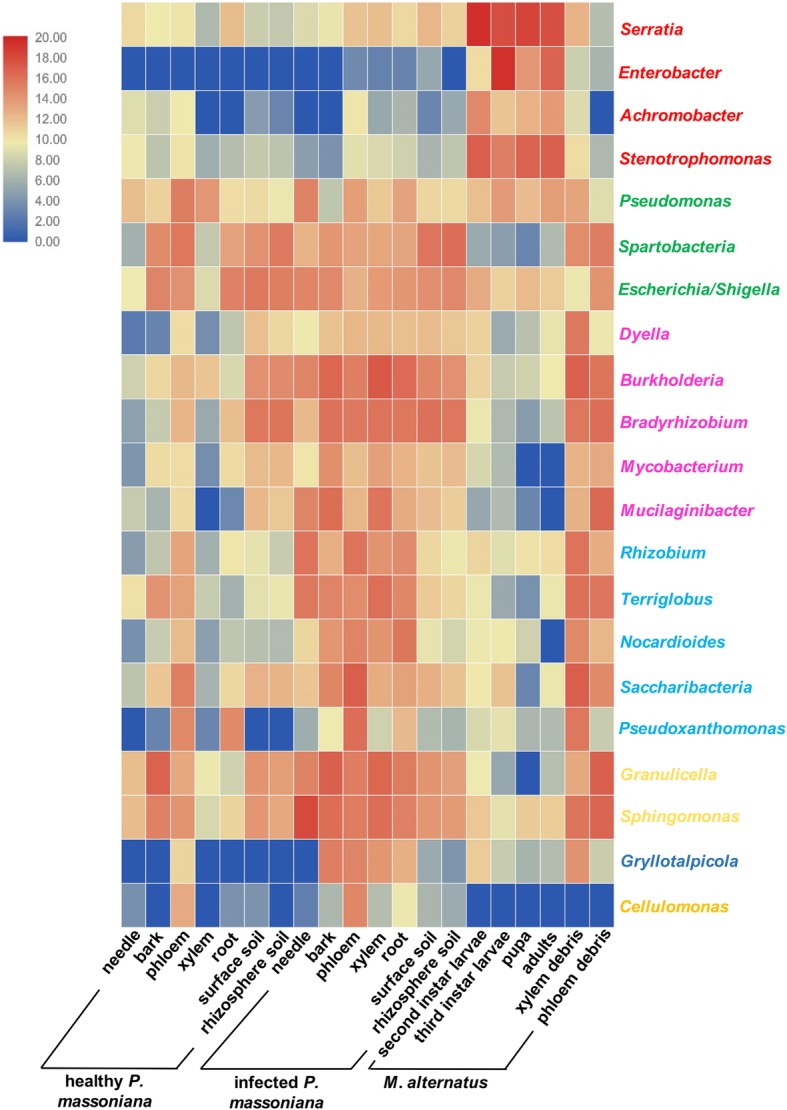
Heatmap of bacterial genera in all samples from *Monochamus alternatus* and infected and healthy *Pinus massoniana*. The red genera were mainly abundant in the *M. alternatus* midgut. The green genera were equally abundant in healthy and infected *P. massoniana*. The pink genera were abundant in the soil of healthy *P. massoniana* and various tissues of infected *P. massoniana*. The light blue genera were abundant in the phloem of healthy *P. massoniana* and various tissues of infected *P. massoniana*. The yellow genera were abundant in the bark of healthy *P. massoniana* and various tissues of infected *P. massoniana*. The dark blue genus was mainly present in the bark, phloem, xylem, and root of infected *P. massoniana*. The orange genus was mainly present in the phloem of *P. massoniana*, and it was more abundant in infected pines than healthy pines

The heatmap shows that genera *Escherichia/Shigella*, *Pseudomonas*, and *Spartobacteria* were mainly distributed in pines, and their overall level was constant in healthy and infected pines (Fig. 4, labeled green). Several bacterial genera were mainly found in the infected pines and soil of healthy pines, including *Dyella*, *Burkholderia*, *Bradyrhizobium*, *Mycobacterium*, and *Mucilaginibacter* (Fig. 4, labeled pink). The genera *Rhizobium*, *Terriglobus*, *Nocardioides*, and *Saccharibacteria* were mainly found in infected pines and the phloem of healthy pines (Fig. 4, labeled light blue). In addition, the genus *Pseudoxanthomonas* was mostly distributed in the phloem and root of healthy pines (14% in both tissues) and infected pines (39% and 2.56%, respectively) (Fig. 4, labeled light blue). *Granulicella* and *Sphingomonas* genera were mainly distributed in the bark of healthy pines compared to the other health pine tissues, and their relative abundances were increased in all infected pines tissues (Fig. 4, labeled yellow). The genus *Gryllotalpicola* was only found in the phloem (0.1%) of healthy pines (rather than any other of the healthy pine tissues), but it was increased in the bark (4.1%), phloem (3.1%), xylem (1.6%) and root (0.6%) in infected pines, and was also found with low relative abundance in the midgut and frass of *M. alternatus* (Fig. 4, labeled orange). Interestingly, the genus *Cellulomonas* was not found in the midgut of *M. alternatus*, and the highest relative abundance occurred in the phloem of infected pines (2.9%), followed by the phloem of healthy *M. alternatus* (0.8%). Its relative abundance was also low (< 0.01%) in the needle, root, and surface soil of healthy pines, as well as in the needle, bark, xylem, root, surface soil, and rhizosphere soil of infected pines (Fig. 4, labeled blue).

### Specific bacterial genera in the midgut of *M. alternatus*

**Fig. 5:**
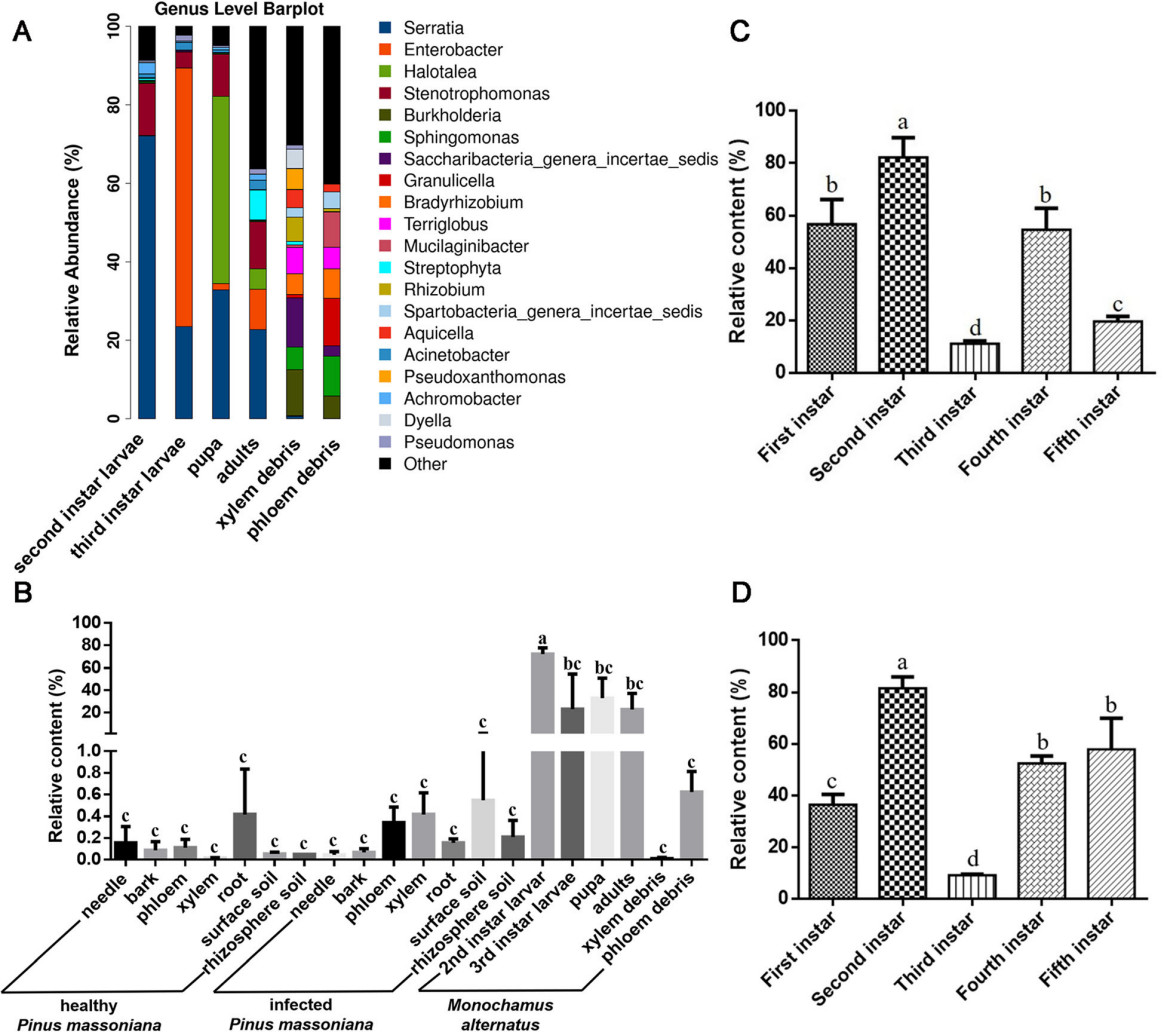
Distribution of the bacterial genus *Serratia* in the midgut of various instar *Monochamus alternatus* larvae. (**a**) Stacked bar plot of bacterial genera in *M. alternatus* samples. (**b**) Distribution of *Serratia* in samples from *M. alternatus* and infected and healthy *Pinus massoniana* (based on sequencing data). Distribution of *Serratia* sp. in the midgut of various instars of (**c**) wild-caught and (**d**) artificially fed *M. alternatus* (based on colony-forming units). a, b, c and d indicate *p* < 0.05

The bacterial genera *Serratia*, *Enterobacter*, *Achromobacter*, and *Stenotrophomonas* were dominant in the midgut of *M. alternatus* (Fig. 4, labeled red). *Serratia* was the most abundant bacterial genus in the midgut of instar II larvae. *Enterobacter* was the most abundant genus in the midgut of instar III larvae (65%), and it was also highly abundant in the midgut of adult insects (10.30%). *Halotalea* was the most abundant bacterial genus in the pupae midgut (47.69%) (Fig. 5a).

Interestingly, the relative abundance of genus *Serratia* was different in various instars of *M. alternatus*. In the habitat niche, *Serratia* was detected in all samples, but with low relative abundance (< 0.5%). However, *Serratia* was enriched in the midgut of *M. alternatus* larvae; it peaked at 72.11% in the instar II larvae, decreased in the instar III larvae (23.46%), increased again in the pupae (32.85%), and was lowest in adults (22.71%). Additionally, *Serratia* was found in the frass of the instar II and III larvae (< 0.6%). These results indicate a close relationship between genus *Serratia* and *M. alternatus* (Fig. 5b).

The colony-forming unit assays showed that *Serratia* sp. was present in midgut of instars I–V regarding both *M. alternatus* reared on an artificial diet and wild-caught *M. alternatus*. *Serratia* sp. peaked in instar II (about 81% in both), was at a minimum in instar III (9% in the larvae reared on the artificial diet and 11% in the wild-caught larvae), and was relatively stable for instars I and IV between the reared on artificial diet and wild-caught groups. However, in instar V (diapause), *Serratia* sp. in larvae reared on the artificial diet was higher than in wild-caught larvae (Fig. 5c and d). The results suggest that food has little effect on the relative abundance of *Serratia* sp. in the midgut of *M. alternatus* larvae, but further research is needed on its abundance pattern and whether it is related to the larval metabolic mechanisms.

The heatmap of Spearman’s rank correlation coefficients at the genus level shows that the relative abundance of *Serratia* was positively correlated with *Stenotrophomonas*, *Gryllotalpicola*, and *Pseudoxanthomonas*, and negatively correlated with Gp1 Gp2 Gp3, *Escherichia/Shigella*, *Burkholderia*. *Bradyrhizobium, Sphingomonas, Granulicella*, and *Mucilaginibacter* (Additional file 1: Figure S8).

## Discussion

This study provides a systematic description of the microbial communities in the midgut of *M. alternatus* and its habitat niche based on 16S rDNA gene amplicon sequencing. Samples were collected during the same time period from the same pine stand to ensure the stability of the microbial composition. And the results of rarefaction curves analysis of all samples showed sampling sufficiency.

Soil microbiomes exhibit extremely rich diversity and research shows that plants and insect microbiomes depend on soil microbiomes [14]. Acidobacteria is one of the most dominant phyla in the soil [36], and it was the predominant bacterial phyla in the surface soil and rhizosphere soil of both healthy and infected pines in this study (including Gp1, Gp2, and Gp3). Many studies have shown that Acidobacteria plays a vital role in the ecosystem, and it has a rich diversity of metabolic and genetic functions [37], as well as making a significant contribution to ecological stability [38]. Acidobacteria are the dominant bacteria in most soils because its optimum pH is low [39], though different subgroups of Acidobacteria have different optimum pH values. For example, the subgroup Gp1 grows best in soil environments with a pH of 4–5.5 [40, 41]. Shi et al. found that pinewood nematode infection changes the physical and chemical properties of the soil and the bacterial community composition and diversity; however, Acidobacteria was the predominant bacteria in nematode-infected soil, which had a lower pH than the uninfected soil [35].

Additionally, the soil and infected pines shared multiple bacterial genera. *Bradyrhizobium*, *Burkholderia*, *Dyella*, *Mycobacterium*, and *Mucilaginibacter* were the predominant bacterial genera in infected pines and the soil of healthy pines. Among them, only genus *Bradyrhizobium* was previously found in the soil of nematode-infected and nematode-uninfected pines [35]. Additionally, studies in various countries have reported that genus *Burkholderia* is found on pinewood nematodes [24, 25, 27].

Moreover, the dominant bacteria in pines changed significantly as a result of the damaged caused by pine wilt disease. The dominant bacterial genera in the infected pines are related to plant growth [42–46] and they can degrade compounds, especially cellulose [47–49]. It has been reported that cellulases played an important role during the nematode progressing inside the plant host [27, 50]. Therefore, the dominant bacterial genera were present in all samples from infected pines, but only a few were found in the midgut of *M. alternatus* and healthy pines. Among them, *Rhizobium*, *Saccharibacteria*, *Terriglobus*, *Nocardioides*, and *Pseudoxanthomonas* were only found in the phloem of healthy pines. Additionally, *Granulicella* and *Sphingomonas* were the main genera in the bark of healthy pines. Previous studies reported that the genera *Pseudomonas* and *Pantoea* and the orders Xanthomonadales, Acidobacteriales, and Rhizobiales are associated with *Pinus* spp. [28, 32, 51], and Sphingomonadales was found in both *P. pinaster* and *M. alternatus* [34]. These results indicate the systemic distributions of bacteria in different versions of the habitat niche of *M. alternatus*.

It has been reported that the genus *Gryllotalpicola* was isolated from the midguts of *Megopis sinica*, *M. alternatus*, and *Reticulitermes speratus*, while the genus *Cellulomonas* was isolated from both the midgut and hindgut of *M. sinica* and *M. alternatus* [52, 53] and from the stem of *P. contorta* and the needles of *Thuja plicata* [29]. In this study, however, *Gryllotalpicola* had a relative abundance of only 0.25% in the midgut of *M. alternatus*, and *Cellulomonas* was not found in the midgut of *M. alternatus*. Both *Gryllotalpicola* and *Cellulomonas* can degrade cellulose [52], which is the main nutrient component in the food of wood-boring insects and plays an important role in the growth and development of pests [54]. Therefore, *Gryllotalpicola* sp. is a potential cellulolytic bacterial species that may promote the feeding of *M. alternatus* on infected pines. Moreover, many soil microorganisms have been used as indicators of soil quality, particularly microorganisms that are resistant to heavy metals and toxic chemicals [55, 56]. Therefore, according to their distribution in infected pines, *Gryllotalpicola* spp. and *Cellulomonas* app. Could as potential microbial markers of pine wilt disease in pines.

There is a strong association among *Serratia* sp., *M. alternatus*, and pinewood nematode. *Serratia* spp. has been isolated from *Pinus* spp. and pinewood nematode in various countries [23, 25–27, 32, 51, 57]. *Serratia* sp. has been shown to be present at a high density in the bacterial community of the thorax (44%) and abdomen (95%) of *M. galloprovincialis* adults [17, 18]. In this study, however, the relative abundance of *Serratia* in the midgut of adults (22%) was lower than the relative abundances reported for the thorax and abdomen in previous research, which may be related to the different *Monochamus* species investigated and the different durations since emerging as adults. Notably, in this study, *Serratia* sp. was found in the midgut of larvae and pupa, at 81% in the midgut of instar II larvae. *Serratia* spp. has strong stability for rapid adaptation to the environment [58, 59]. *S. marcescens* PWN146 has been shown to be able to colonize plants [33]. As *Serratia* sp. was present in the habitat niche and enriched in the midgut of *M. alternatus* in this study, *Serratia* sp. (carrying toxins) will likely be able to enter *M. alternatus* larvae.

Additionally, *S. marcescens* has multiple roles after colonizing plants. Under environmental stimulation, it can change from a beneficial bacteria (promoting plant growth) to a plant pathogen [60, 61]. Also, *Serratia* sp. A88copa13 encodes extracellular serralysin and serine proteases [62] and *Serratia* sp. associated with the pinewood nematode can degrade cellulose, which is beneficial for colonization of wood tissues [26]. Moreover, *Serratia* sp., which in the gut of *Dendroctonus armandi* larvae and *M. alternatus* larvae, secretes cellulase and other extracellular enzymes [63, 64]. The main enzymes involved in cellulose depolymerization are endoglucanase, exoglucanase, and β-glucosidase [65, 66]. Many endoglucanases from *Serratia* spp. have been annotated [67–69], and *Serratia* spp. can synthesize β-xylosidase and lignins [64, 70]. Thus, *Serratia* sp. may be a cellulolytic and hemicellulolytic bacteria that can survive in the midgut of *M. alternatus* and its habitat niche.

As mentioned earlier, the main problem associated with using microbial insecticides to control *M. alternatus* is that it is difficult for the insecticides to enter the tree trunk and reach the *M. alternatus* larvae [7–10]. In this study, as an environmental microorganism, the genus *Serratia* was not only present in the healthy pines and soil but it was also enriched in the midgut of *M. alternatus* as a predominant symbiotic bacterial genus. Therefore, genetically engineered *Serratia* sp. could be an ideal agent for expressing insecticidal protein in *M. alternatus* midguts, which would represent a new use of microbes to control *M. alternatus*. Furthermore, the cellulose-degrading bacterial genera *Stenotrophomonas*, *Gryllotalpicola*, and *Pseudoxanthomonas* [49, 52] were positively correlated with *Serratia*. In contrast, the genera Gp1, Gp2, Gp3, *Saccharibacteria, Escherichia/Shigella*, *Bradyrhizobium, Sphingomonas, Terriglobus*, *Burkholderia*, and *Mucilaginibacter* were negatively correlated with *Serratia*, and they are associated with soil pH, plant growth, and cellulose degradation [39, 42, 44, 45]. These bacterial genera provide possible tools for regulating the abundance of *Serratia* sp. in the habitat niche of *M. alternatus*.

## Conclusions

**Fig. 6:**
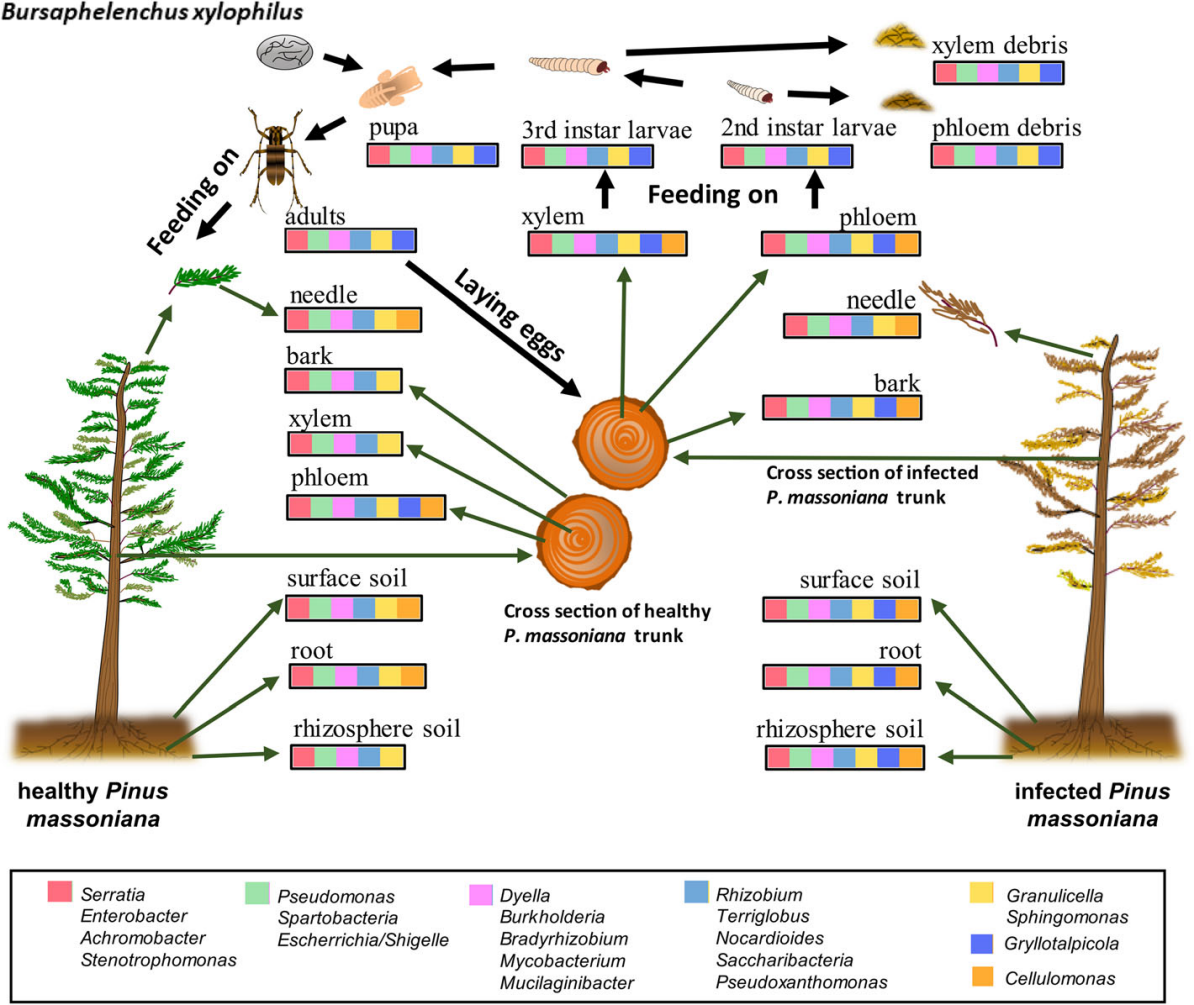
Changes in the distribution of major bacterial groups during the transmission of pinewood nematode by *Monochamus alternatus.* The bacteria are classified into seven color-coded groups according to their distribution in the various samples, as explained in the legend of Fig. 4

This study indicates that the bacterial diversity was significantly increased in infected pines compared to healthy pines. The bacteria detected in this study might play a role in the soil–pines–*M. alternatus* system. *Bradyrhizobium*, *Burkholderia*, *Dyella*, *Mycobacterium*, and *Mucilaginibacter* were dominant bacterial genera in soil and infected pines; *Gryllotalpicola* and *Cellulomonas* were predominant genera in infected pines; and the genus *Serratia* was present in the habitat niche and was enriched in the midgut of *M. alternatus* (Fig. 6). Systematic analysis of the microbiomes in *M. alternatus* and its habitat niche is important not only to better understand the role of bacteria in pine wilt disease but also to provide a new strategy for the control of pine wilt disease.

## Methods

### Sample collection

All samples were collected from a pine stand (26° 9′ 1.6″ N, 119° 35′ 33″ E) in Guantou city, Lianjiang county, Fujian province, China. The *P. massoniana* (pines) were 25 years old and had not been damaged by other diseases or insects besides pine wilt disease. To determine whether the trees were infected with pinewood nematode, their trunks were cut into cross-sections to assess the presence of *M. alternatus* or other insects, and the Baermann funnel method followed by polymerase chain reaction (PCR) with specific primers was then used to confirm the presence of the pinewood nematode [71]. Thereafter, three healthy and three infected pine trees (10 m away from each other) were selected. For both the infected and healthy pines, secondary branches were sampled, which involved collecting needles, bark, phloem, and xylem. Regarding the surface soil, rhizosphere soil, and roots, samples were obtained from points in the same direction as the sampled secondary branches of the infected and healthy pines. After removing leaves and roots from the surface soil, soil samples were collected at each sampling point with a soil auger at a depth of 0–5 cm (for surface soil) and a depth of 5–15 cm (for rhizosphere soil). Roots samples were isolated from the rhizosphere soils. Samples (with three replicates for each samples type) were placed in separate sterile plastic containers. Next, three instar II larvae, three instar III larvae, and three pupae were collected from the logs, which had the entry-points of *M. alternatus*, obtained from three infected pines. The *M. alternatus* instars were determined by head capsule width [72, 73]. Thereafter, other logs obtained from the infected trees were placed in cages near the sampling points and a single *M. alternatus* adult was obtained as it emerged from the log at each sampling point. The instar II and III larvae were maintained in a plastic box and their frass was also collected. All samples were placed in dry ice immediately after collection, brought back to the laboratory, and then stored at − 80 °C until use.

### DNA extraction

Microbial DNA was extracted from each sample by mechanical lysis in sodium dodecyl sulfate (SDS), followed by treatment with hexadecyl trimethyl ammonium bromide (CTAB) [74]. Pine samples (1.0 g) and soil samples (0.3 g) were homogenized with liquid nitrogen and mixed with 0.9 mL DNA extraction buffer (100 mM Tris-HCl [pH 8.0], 25 mM sodium ethylenediaminetetraacetic acid [EDTA, pH 8.0], 10% SDS, 0.5 M NaCl, and 1% CTAB) and 5 μL proteinase K (10 mg/mL) in 1.5-mL tubes followed by horizontal shaking at 230 rpm for 30 min at 37 °C. Thereafter, 0.3 mL of 20% SDS was added, and the samples were incubated at 65 °C for 2 h with gentle end-over-end inversion every 20 min. The samples were frozen at − 70 °C for 20 min and then incubated at 65 °C for 20 min, and this process was repeated three times. The samples were then centrifuged at 6000×g for 10 min at 4 °C and the supernatants were transferred into 50-mL centrifuge tubes. Supernatants from two cycles of extractions were combined and mixed with an equal volume of phenol: chloroform: isoamyl alcohol (25:24:1, v/v/v). The aqueous phase was recovered after centrifugation, and DNA was precipitated using 0.1 volume of sodium acetate and 0.6 volume of isopropanol at room temperature for 1 h. A DNA pellet was obtained by centrifugation at 14,000×g for 30 min at room temperature, washed twice with cold 70% ethanol, and resuspended in sterile deionized water.

Each insect surface was sterilized with 70% ethanol for 1 min, and then rinsed with sterile water. Midgut samples were dissected under a stereoscopic microscope and homogenized in 500 μL Tris-EDTA (TE) buffer. Microbial DNA was extracted from each midgut sample using an E.Z.N.A.® Bacteria DNA Kit (Omega Bio-Tek, Norcross, GA, USA). All DNA samples were stored at − 20 °C until further use.

### 16S rDNA gene amplicon sequencing

The 16S rDNA gene was amplified using a KAPA HiFi Hotstart ReadyMix PCR kit (Kapa Biosystems, Boston, Massachusetts, USA) and the universal primers 341F/806R (341F: ACTCCTACGGGRSGCAGCAG, 806R: GGACTACVVGGGTATCTAATC) targeting the V3–V4 region. PCR amplicons were purified using an AxyPrep DNA kit (Axygen Biosciences, Central Avenue, Union City, CA, USA) and quantified using a Qubit 2.0 Fluorometer (Thermo Fisher Scientific, Waltham, MA, USA). The DNA was pooled, with a final concentration of 10 ng/μL. The quality of the amplicon libraries was assessed using a NanoDrop 2000 UV spectrophotometer (Thermo Fisher Scientific) and by agarose gel electrophoresis. The amplicon library sequencing was performed on an Illumina HiSeq PE250 platform (Illumina, San Diego, CA, USA) according to the standard protocols at RealBio Technology, Shanghai, China.

### Bioinformatics analysis

The paired-end reads were merged into longer tags and quality filtered using PANDAseq to obtain high-quality tags [75]. Amplicon libraries were sequenced by paired-end reads of 425 bp. After quality control, OTUs were clustered with a similarity cutoff of 97% using Usearch [76]. The OTUs were further subjected to a taxonomy-based analysis using the Ribosomal Database Project (RDP) algorithm and the Greengenes database (http://greengenes.lbl.gov) [77]. Alpha diversity (Shannon index) and beta diversity (weighted UniFrac, principal coordinate analysis [PcoA]) were analyzed using QIIME [78]. LEfSe analyses were performed using an online LEfSe tool (http://huttenhower.sph.harvard.edu/galaxy) [79]. A heatmap of Spearman’s rank correlation coefficients (regarding the relative abundances of the bacteria genera in all samples) was constructed using the corrplot package in R.

### Colony-forming unit assays of *Serratia* sp. in *M. alternatus* midgut

The relative abundances of *Serratia* sp. in the midgut of *M. alternatus* instars I–V were analyzed by counting the colony-forming units [28, 80]. The instars were reared on an artificial diet (wheat bran 60 g, shrimp shell powder 10 g, sorbate 2 g, sodium benzoate 4 g, yeast 25 g, agar 30 g, phloem powder 100 g, xylem powder 50 g, sucrose 40 g, and water 300 mL) and the second generation of larvae were used for the experiments. Additionally, wild-caught instars I–V were collected from the abovementioned pine stand. There were 10 replicates for each instar in the artificial diet and wild-caught groups. 16S rDNA sequencing and physiological and biochemical analysis indicated that the *Serratia* sp. in the midgut of *M. alternatus* was *S. marcescen*s. Each midgut sample was dissected under a stereoscopic microscope, homogenized in 100 μL TE buffer for 3 min, and stored on ice until use. The homogenate was diluted with TE buffer and plated on *Serratia* Differential Medium (HIMEDIA, India) and Luria–Bertani medium. *Serratia* Differential Medium was used to differentiate between *S. marcescens*, *S. rubidaea*, and *S. liquefaciens*, based on their ability to ferment L-arabinose and decarboxylate ornithine [81]. The data were organized using Microsoft Excel 2016 and potential significant differences were analyzed by Student’s t-tests and analysis of variance (ANOVA) in SPSS 18.0. A *p*-value of < 0.05 was considered statistically significant.

## Supplementary information

**Additional file 1: Figure S1.** Rarefaction curves analysis of samples from *Monochamus alternatus* and its habitat niche. **Figure S2.** Profiling barplot of bacterial phyla from *Monochamus alternatus* and its habitat niche. **Figure S3.** The tax tree of bacterial genera from infected *Pinus massoniana*. **Figure S4.** The tax tree of bacterial genera from *Monochamus alternatus*. **Figure S5.** The tax tree of bacterial genera in the process of instar II larvae of *Monochamus alternatus* feeding on the phloem. **Figure S6.** The tax tree of bacterial genera in the process of instar III larvae of *Monochamus alternatus* feeding on the xylem. **Figure S7.** The tax tree of bacterial genera in the process of *Monochamus alternatus* adults feeding on the bark. **Figure S8.** The heatmap of Spearman’s rank correlation coefficients of bacterial genera.

## Data Availability

All raw sequences were deposited in the National Center for Biotechnology Information (NCBI) Sequence Read Archive (BioProject: PRJNA561715).

## Abbreviations

ANOVA: Analysis of variance
CTAB: Hexadecyl trimethyl ammonium bromide
EDTA: Ethylenediaminetetraacetic acid
LEfSe: Linear discriminant analysis effect size
NCBI: National Center for Biotechnology Information
OTU: Operational taxonomic unit
RDP: Ribosomal Database Project
SDS: Sodium dodecyl sulfate
TE: Tris-EDTA
