# Supplementary material for: Characterization of bacterial communities associated with the pinewood nematode insect vector Monochamus alternatus Hope and the host tree Pinus massoniana
Source: BMC Genomics. 2020 May 1;21:337. doi: 10.1186/s12864-020-6718-6 (PMC7195709; doi:10.1186/s12864-020-6718-6)
Supplement: Supplementary file 1 — Additional file 1: Figure S1. Rarefaction curves analysis of samples from Monochamus alternatus and its habitat niche. Figure S2. Profiling barplot of bacterial phyla from Monochamus alternatus and its habitat niche. Figure S3. The tax tree of bacterial genera from infected Pinus massoniana. Figure S4. The tax tree of bacterial genera from Monochamus alternatus. Figure S5. The tax tree of bacterial genera in the process of instar II larvae of Monochamus alternatus feeding on the phloem. Figure S6. The tax tree of bacterial genera in the process of instar III larvae of Monochamus alternatus feeding on the xylem. Figure S7. The tax tree of bacterial genera in the process of Monochamus alternatus adults feeding on the bark. Figure S8. The heatmap of Spearman’s rank correlation coefficients of bacterial genera. [file 12864_2020_6718_MOESM1_ESM.docx]

**Additional file1**


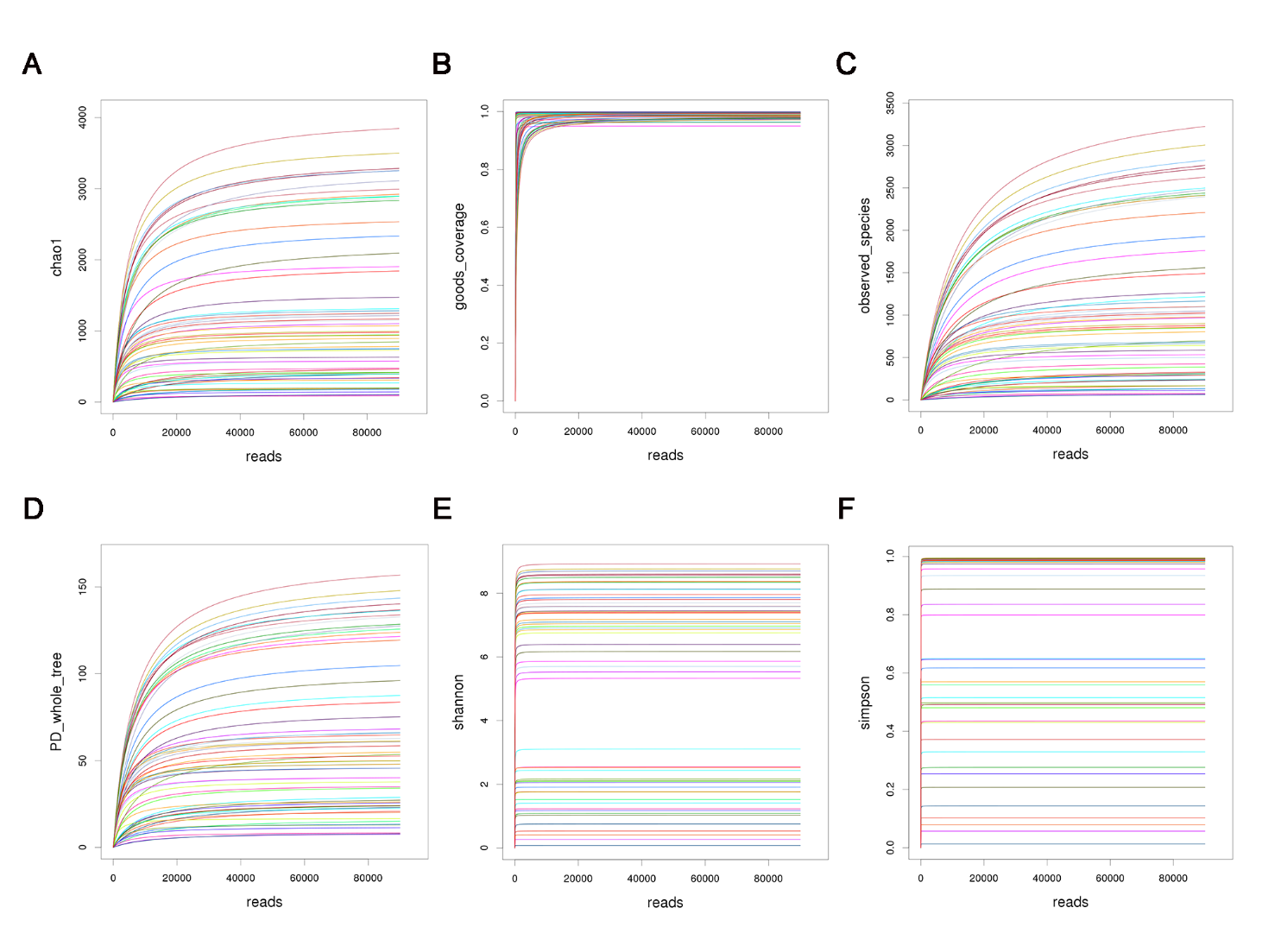


**Figure S1. Rarefaction curves analysis of samples from *Monochamus alternatus* and its habitat niche.** (A) Chao1 index, (B) goods coverage index, (C) observed species index, (D) PD_whole_tree index (E) Shannon index, (F) Simpson index. According to the rarefaction curves, the number of sequences was able to reflect the main bacterial information in each sample.


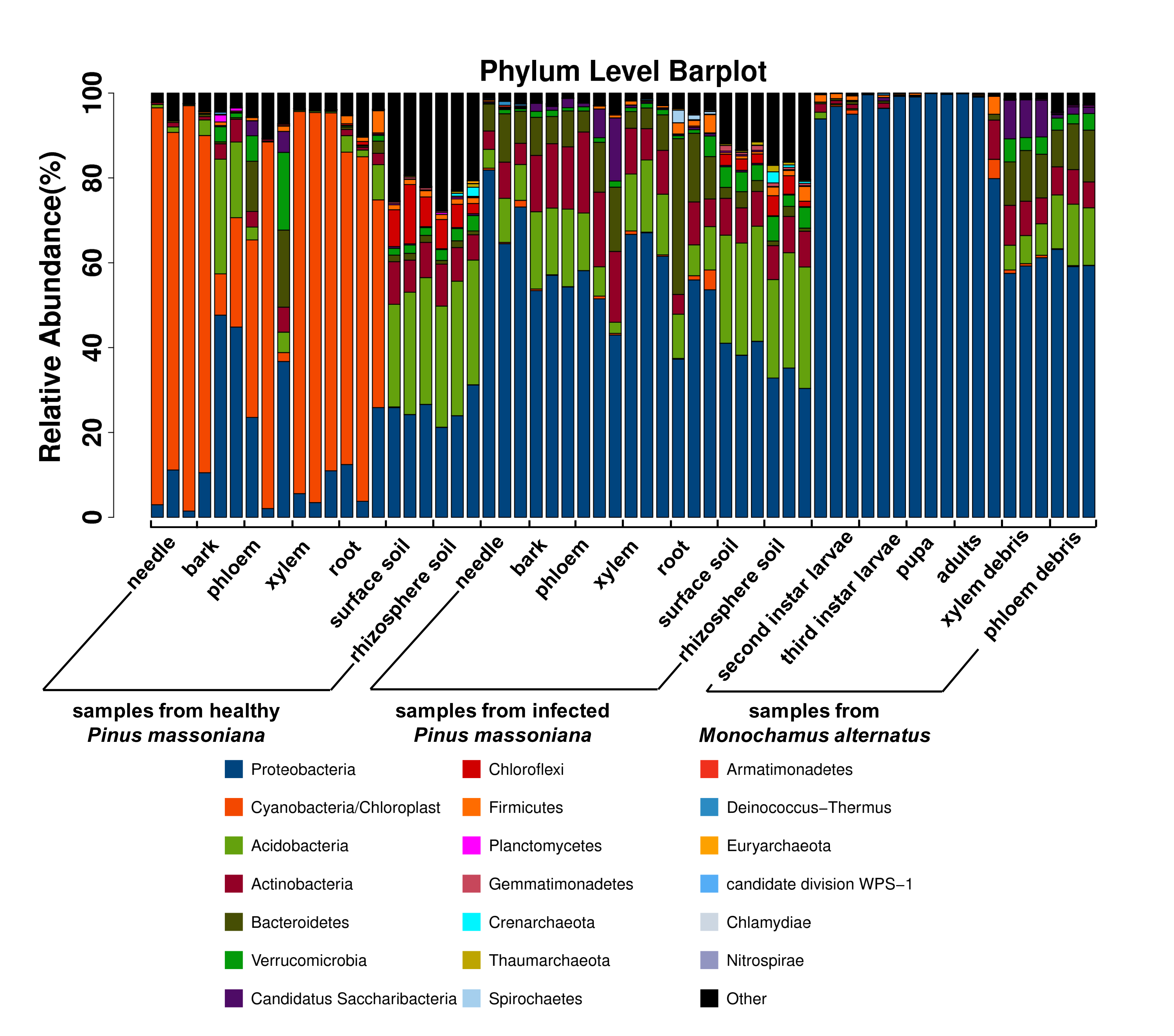


**Figure S2. Profiling barplot of bacterial phyla from *Monochamus alternatus* and its habitat niche.** Only the 20 most abundant OTUs are represented. The remaining microbiota are included in the group “other”.


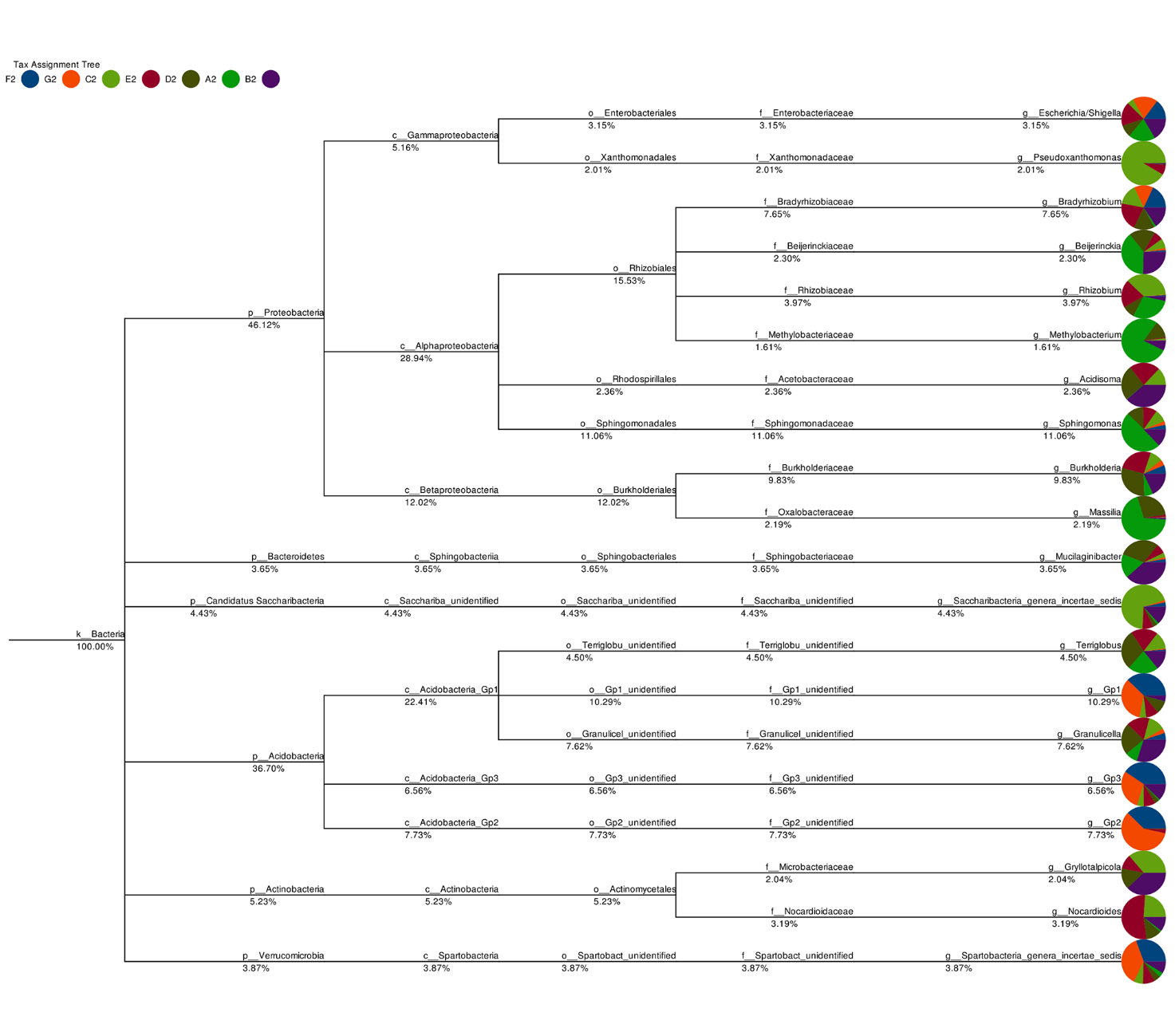


**Figure S3. The tax tree of bacterial genera from infected** [***Pinus***](javascript:;) [***massoniana***](javascript:;)**.** A2: infected pine needles, B2: infected pine barks, C2: infected pine phloem, D2: infected pine xylem, E2: infected pine root, F2: infected pine surface soil, G2: infected pine rhizosphere soil.


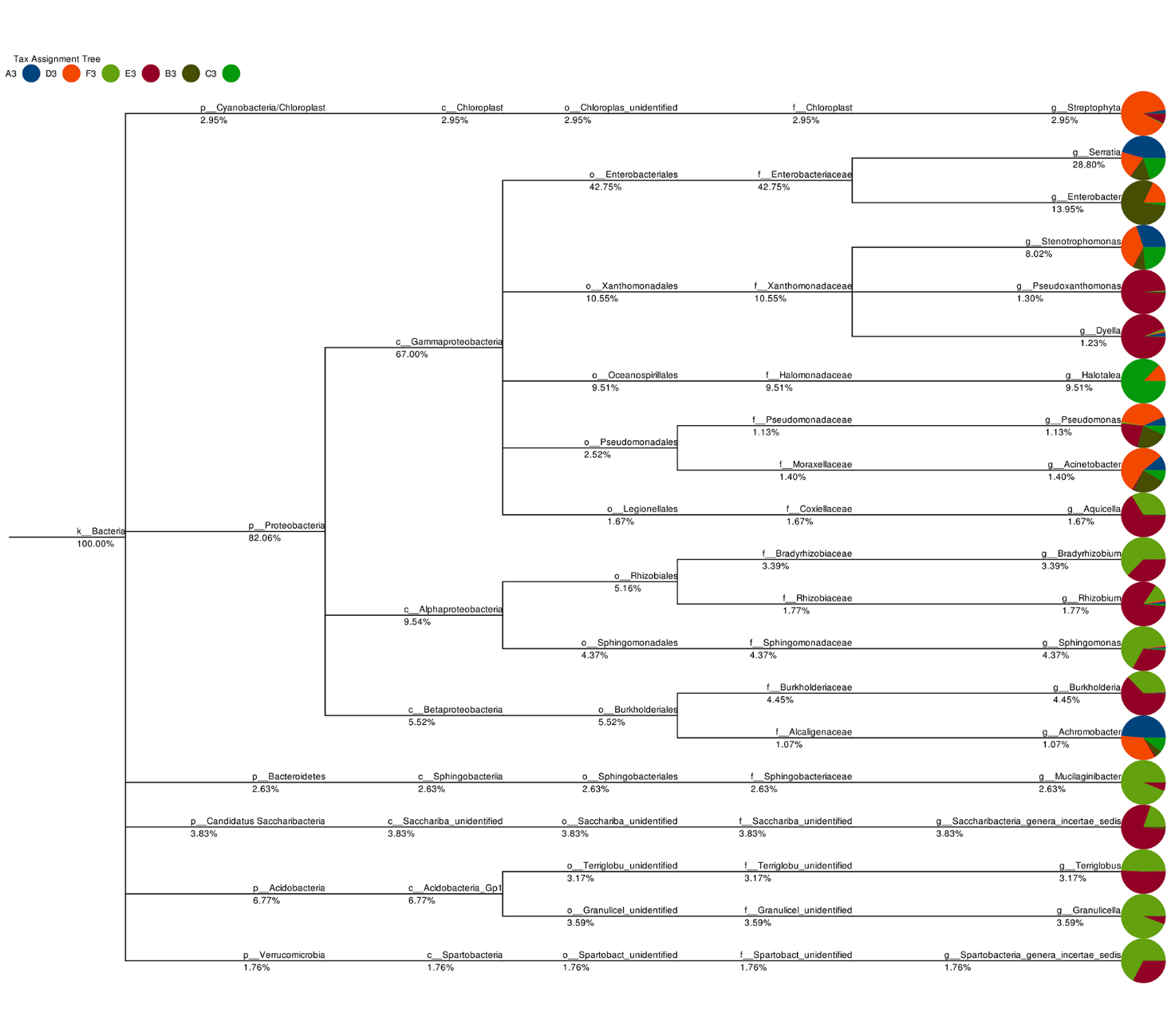


**Figure S4. The tax tree of bacterial genera from *Monochamus alternatus*.** A3: instar II larvae midgut, B3: instar III larvae midgut, C3: pupa midgut, D3: adult midgut, E3: frass of instar III larvae, F3: frass of instar II larvae.


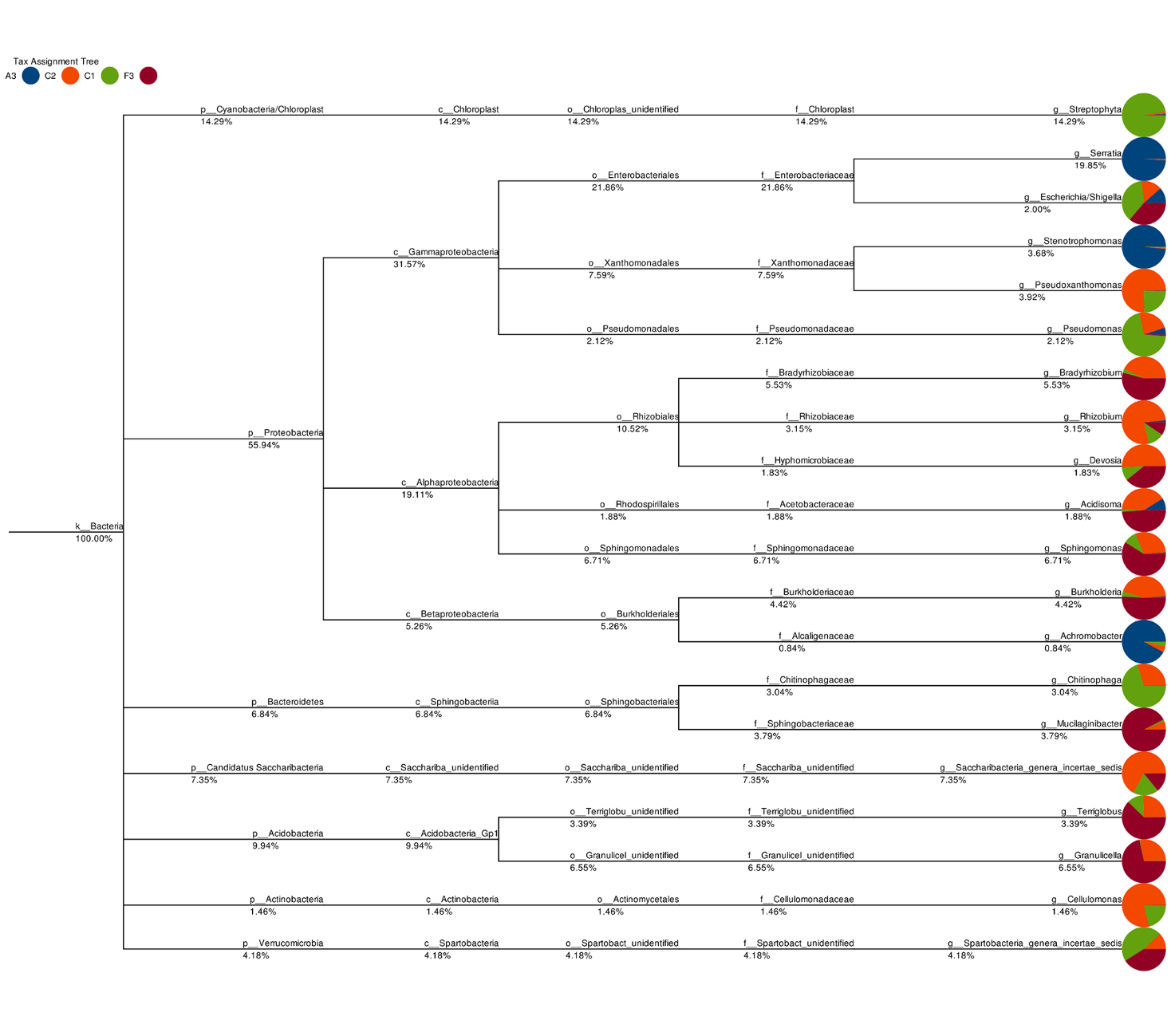


**Figure S5. The tax tree of bacterial genera in the process of instar II larvae of *Monochamus alternatus* feeding on the phloem.** A3: instar II larvae midgut, C1: healthy pine phloem, C2: infected pine phloem, F3: frass of instar II larvae.


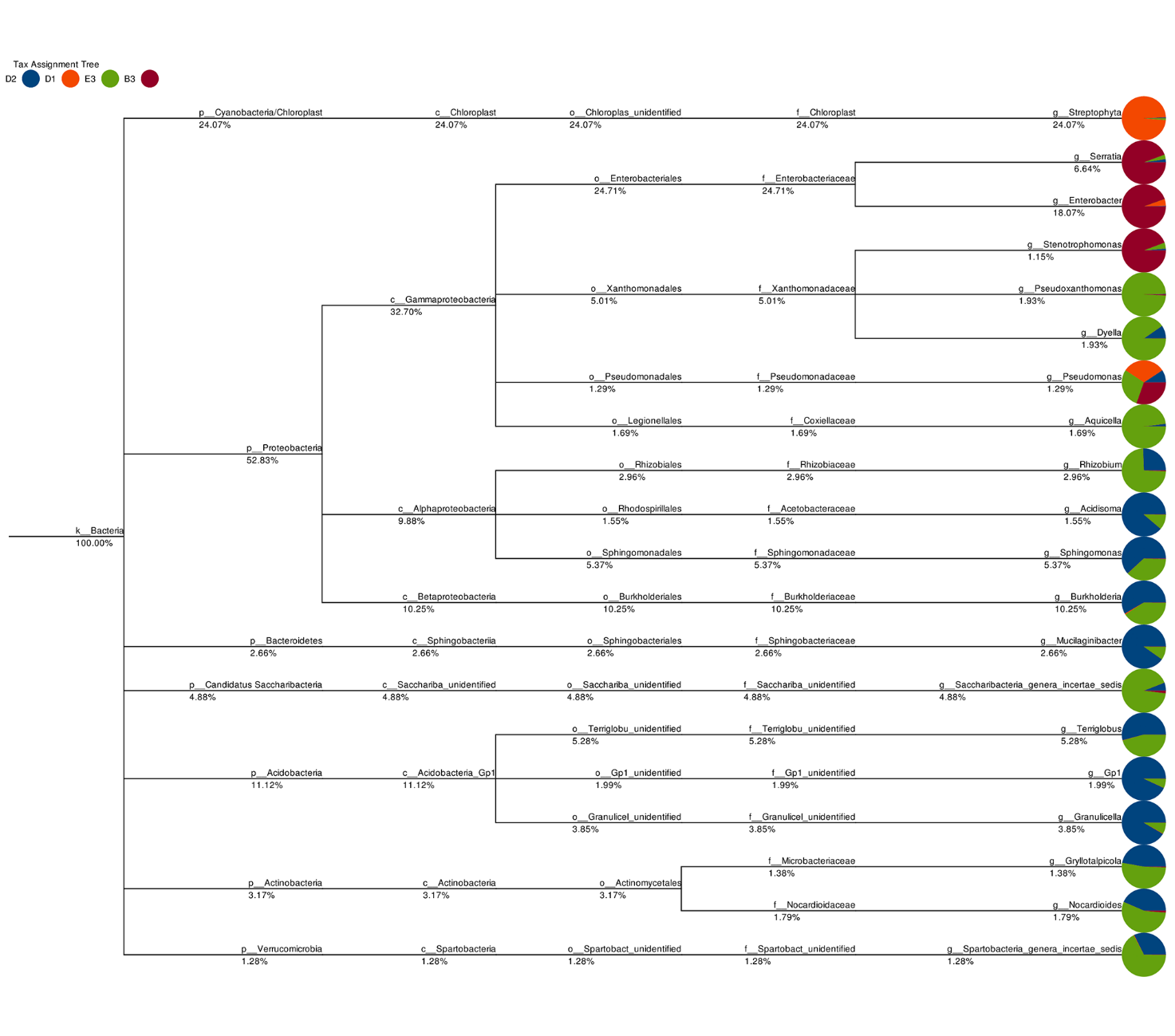


**Figure S6. The tax tree of bacterial genera in the process of instar III larvae of *Monochamus alternatus* feeding on the xylem.** B3: instar III larvae midgut, D1: healthy pine xylem, D2: infected pine xylem, E3: frass of instar III larvae.


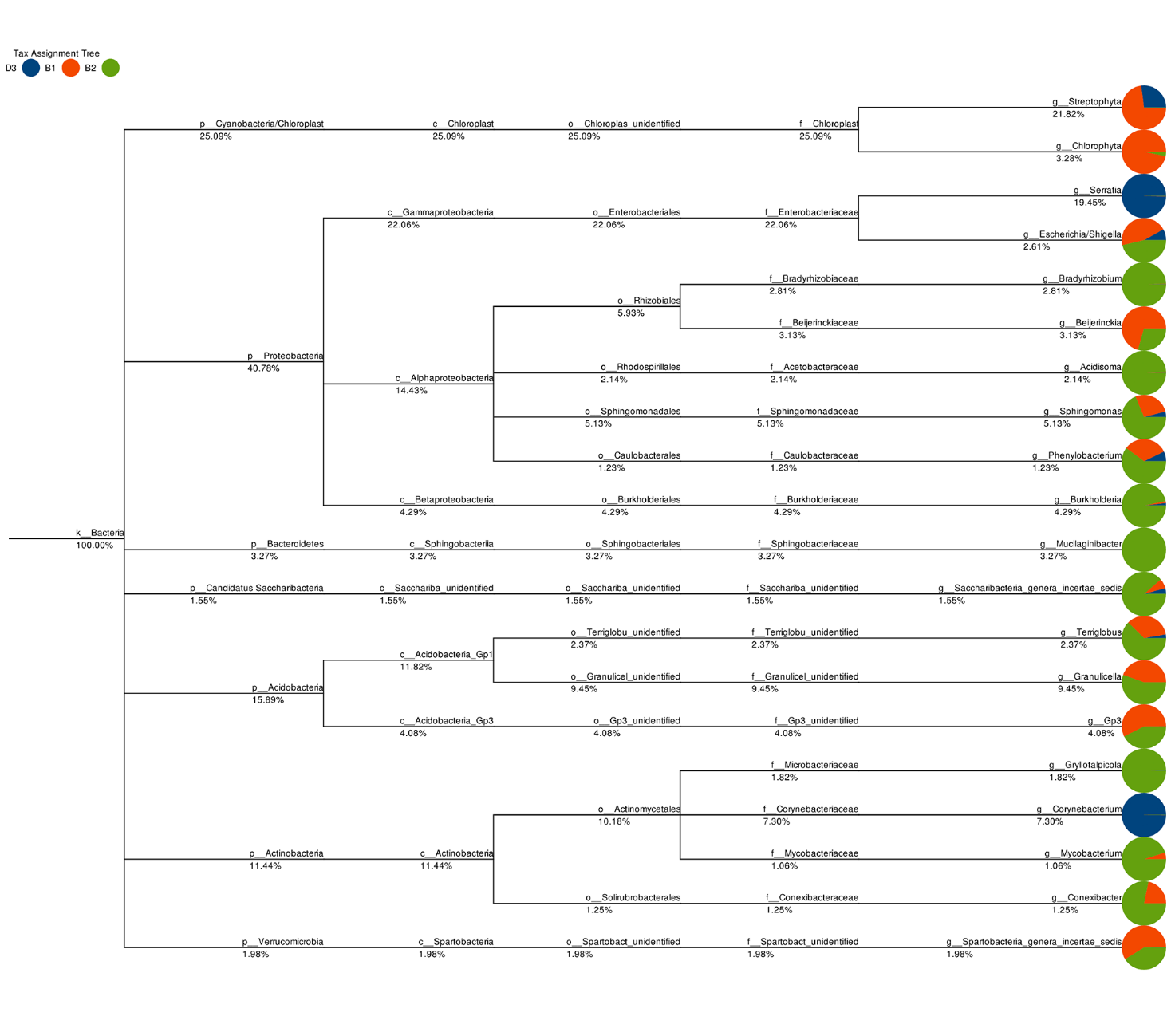


**Figure S7. The tax tree of bacterial genera in the process of *Monochamus alternatus* adults feeding on the bark.** D3: adult midgut, B1: healthy pine barks. B2: infected pine barks.


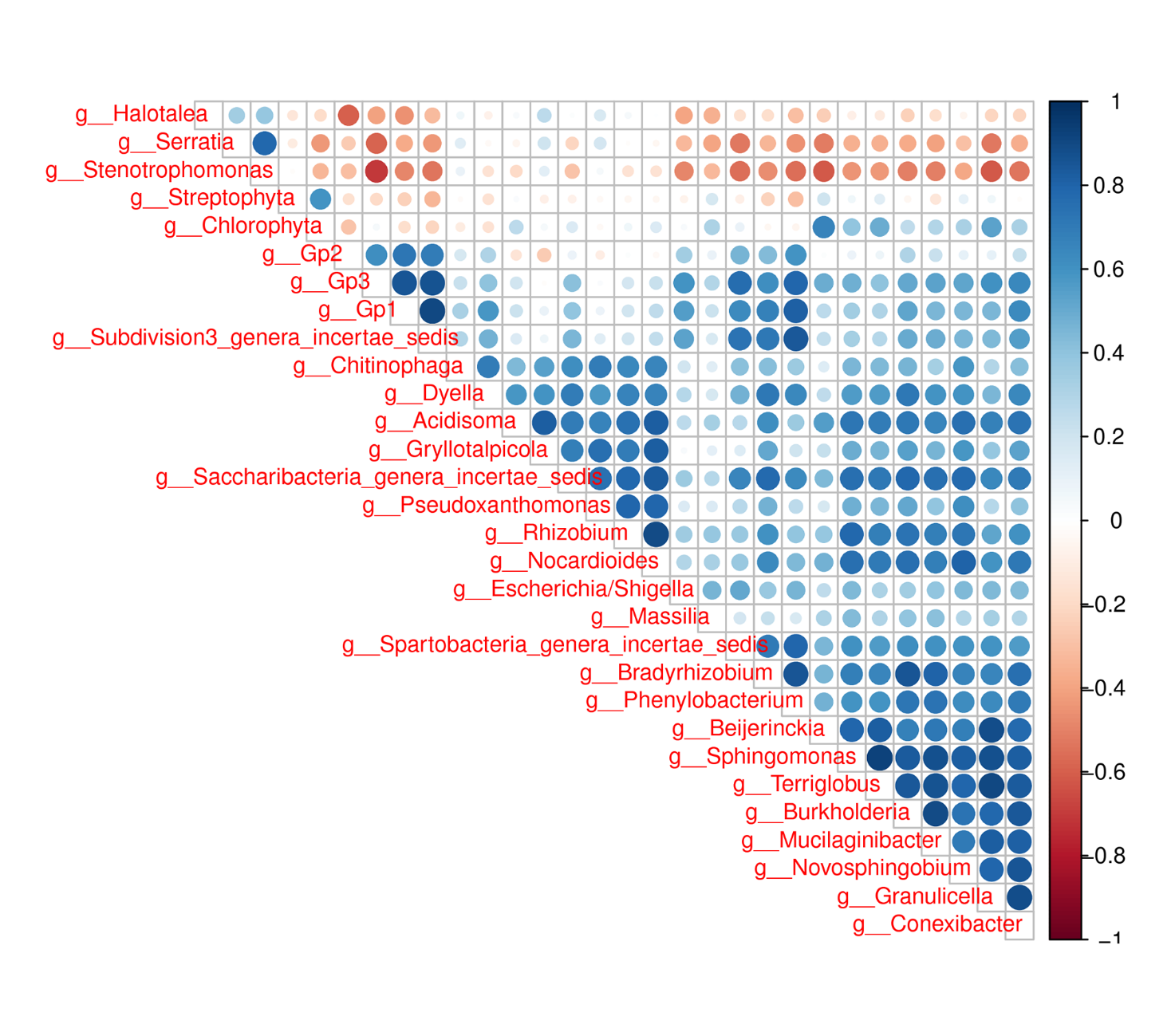


**Figure S8. The heatmap of Spearman’s rank correlation coefficients of bacterial genera.** In genus level, the difference species were obtained by rank sum test, and the relationship between the difference species in the first 30 abundance was calculated. Blue means positive correlation and red means negative correlation. The darker the color, the stronger the correlation between species.
